# Clifton Hall

**Published:** 1861-09

**Authors:** 


CLIFTON HALL.—A number of physicians of Philadelphia, by invitation of Dr. Given, visited the private lunatic asylum of this gentleman, at Clifton Hall, Media, in this State, on the 13th ultimo; and, after a careful inspection of its wards and grounds, adopted a series of resolutions highly complimentary to the character of the establishment, the manner in which it is conducted, and its eligibility as a place of resort for the insane. We regret that want of space prevents the insertion of the resolutions.

